# (*Z*)-*tert*-Butyl 2-(4-amino-9*H*-fluoren-9-yl­idene)acetate

**DOI:** 10.1107/S1600536808029735

**Published:** 2008-09-20

**Authors:** Marios Krokidis, Dionissios Papaioannou, Vassilios Nastopoulos

**Affiliations:** a Department of Chemistry, University of Patras, 265 04 Patras, Greece

## Abstract

The title compound, C_19_H_19_NO_2_, obtained as an almost equimolar mixture (as shown by ^1^H NMR) with the *E* isomer through a Wittig reaction between 4-amino-9*H*-fluoren-9-one and the stabilized ylide Ph_3_P=CHCO_2_C(CH_3_)_3_, was obtained pure in the *Z* configuration following crystallization from toluene. The mol­ecule shows a planar arrangement of the ring system and the new double bond, whereas the carbonyl O atom forms a 45.1 (3)° dihedral angle with it. The mol­ecules are linked by N—H⋯O hydrogen bonds, forming cyclic structures with *R*_4_^4^(24) graph-set motifs. These motifs are connected to each other, giving rise to a sheet structure parallel to the *ab* plane. The linkage within the sheets is further enhanced by π–π stacking inter­actions between the fluorene units [centroid–centroid distance = 3.583 (2) Å].

## Related literature

For general background on retinoids, see: Meyer *et al.* (1978 ▶); Sporn *et al.* (1994 ▶). For hydrogen-bond motifs, see: Bernstein *et al.* (1995 ▶). For related literature, see: Magoulas & Papaioannou (2003 ▶).
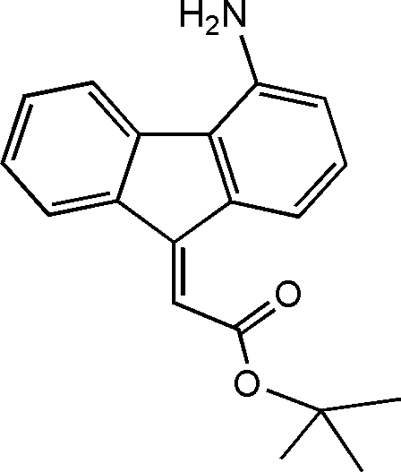


## Experimental

### 

#### Crystal data

C_19_H_19_NO_2_
*M* *_r_* = 293.35
Orthorhombic, 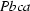

*a* = 9.0820 (12) Å
*b* = 13.7330 (17) Å
*c* = 24.568 (3) Å
*V* = 3064.2 (7) Å^3^
*Z* = 8
Mo *K*α radiation
μ = 0.08 mm^−1^
*T* = 100 (2) K
0.32 × 0.26 × 0.16 mm

#### Data collection

Oxford Diffraction Xcalibur-3 with Sapphire CCD diffractometer
Absorption correction: multi-scan (*CrysAlis RED*; Oxford Diffraction, 2008 ▶) *T*_min_ = 0.956, *T*_max_ = 0.989
18331 measured reflections
2671 independent reflections
1671 reflections with *I* > 2σ(*I*)
*R*_int_ = 0.119

#### Refinement

*R*[*F* ^2^ > 2σ(*F* ^2^)] = 0.063
*wR*(*F* ^2^) = 0.143
*S* = 1.01
2671 reflections
211 parameters
H atoms treated by a mixture of independent and constrained refinement
Δρ_max_ = 0.30 e Å^−3^
Δρ_min_ = −0.22 e Å^−3^

### 

Data collection: *CrysAlis CCD* (Oxford Diffraction, 2008 ▶); cell refinement: *CrysAlis RED* (Oxford Diffraction, 2008 ▶); data reduction: *CrysAlis RED*; program(s) used to solve structure: *SIR92* (Altomare *et al.*, 1994 ▶); program(s) used to refine structure: *SHELXL97* (Sheldrick, 2008 ▶); molecular graphics: *PLATON* (Spek, 2003 ▶), *ORTEP-3* (Farrugia, 1997 ▶) and *DIAMOND* (Brandenburg, 2008 ▶); software used to prepare material for publication: *WinGX* (Farrugia, 1999 ▶) and *publCIF* (Westrip, 2008 ▶).

## Supplementary Material

Crystal structure: contains datablocks I, global. DOI: 10.1107/S1600536808029735/dn2370sup1.cif

Structure factors: contains datablocks I. DOI: 10.1107/S1600536808029735/dn2370Isup2.hkl

Additional supplementary materials:  crystallographic information; 3D view; checkCIF report

## Figures and Tables

**Table 1:** Hydrogen-bond geometry (Å, °)

*D*—H⋯*A*	*D*—H	H⋯*A*	*D*⋯*A*	*D*—H⋯*A*
N—H1N⋯O1^i^	0.85 (3)	2.62 (3)	3.295 (3)	137 (3)
N—H2N⋯O2^ii^	0.92 (3)	2.24 (3)	3.133 (3)	163 (3)

Symmetry codes: (i) 
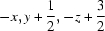
; (ii) 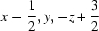.

**Fig. 1.**
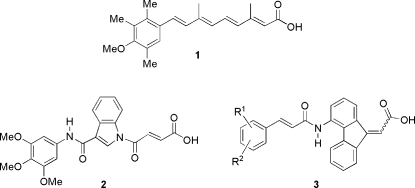
Synthetic scheme, part 1.

**Fig. 2.**
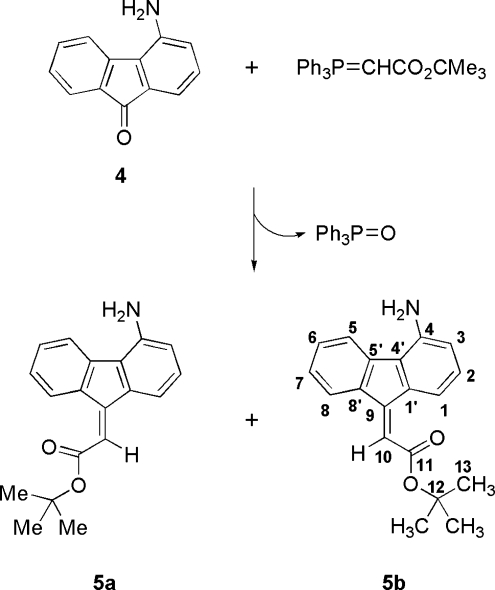
Synthetic scheme, part 2.

**Fig. 3.**
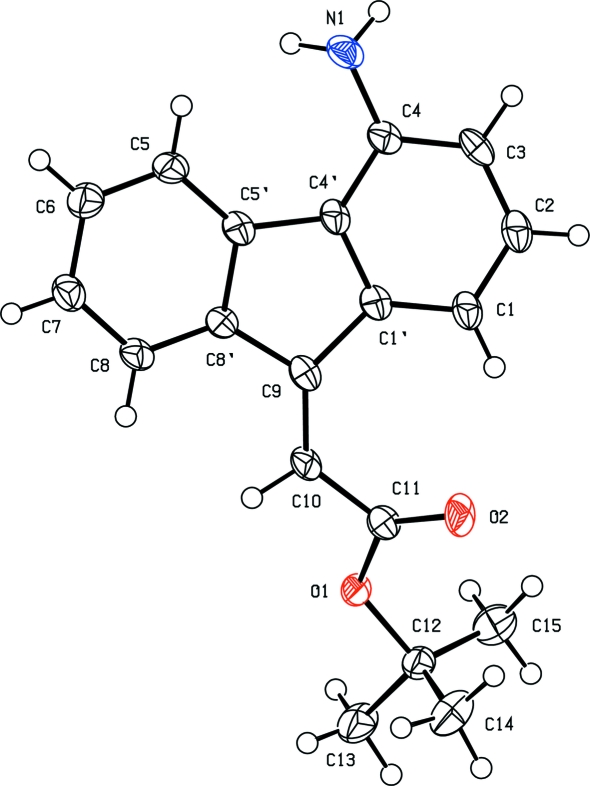
The molecular structure of the title compound (5 b) with the atom-labelling scheme. Displacement ellipsoids are drawn at the 50% probability level.

**Fig. 4.**
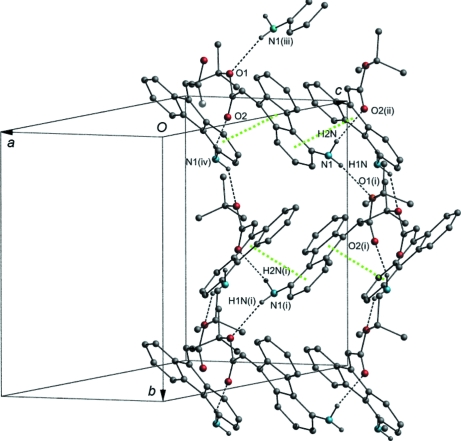
Part of the crystal structure of compound 5b, showing the network of the connected R44(24) cyclic structures parallel to the ab plane and the π—πstacking interactions among the fluorene moieties (green dotted lines). For the sake of clarity, only the H-atoms involved in the bonding pattern (dashed lines) are drawn. [Symmetry codes: (i) -x, y+1/2, -z+3/2, (ii) x-1/2, y, -z+3/2, (iii) -x, -0.5+y, 1.5-z and (iv) 0.5+x, y, 1.5-z]

## supplementary crystallographic information

### Comment

Retinoids, a large family of natural and synthetic compounds structurally related to vitamin A play an important role in a variety of biological functions including vision, development, reproduction and cell differentiation and have been applied successfully to the management of severe skin disorders (Sporn *et al.*, 1994; Meyer *et al.*, 1978). For example, acitretin (1) is presently regarded as the drug of choice for the treatment of psoriasis. However, retinoids are toxic compounds in large doses as well as teratogenic. Therefore, a huge array of analogs have been synthesized aiming at improving the therapeutic efficacy to toxicity index as well as to secure better selectivities for various therapeutic applications. These analogs usually involve changes in the lipophilic part of the molecules and/or the tetraene chain. As concerns the latter, double bonds have been for example replaced by the isosteric amide bond and/or incorporated into aromatic rings to restrict conformational freedom of the chain (Sporn *et al.*., 1994). Along this line, we have recently reported the synthesis of analogs like compound 2 (Magoulas & Papaioannou, 2003). We thought that the tetraene chain might be mimicked by compounds of the general formula 3, which could be readily assembled by joining commercially available cinnamic acids and an 4-amino-9*H*-fluoren-9-one derived α,β-unsaturated carboxylic acid (Fig. 1).

The latter could be readily obtained by a Wittig reaction between 4-amino-9*H*-fluoeren-9-one (4) and the stabilized ylide *tert*-butoxycarbonylmethylenetriphenylphosphorane(BCMP). Indeed, condensation of 4 and BCMP, followed by routine flash column chromatography purification of the reaction mixture, provided *tert*-butyl 2-(4-amino-9*H*-fluoren-9-ylidene)acetate as an inseparable, by TLC, mixture of the E (5a) and *Z* (5 b) isomers (Fig. 2).

Examination of this reaction product by ^1^H-NMR revealed that the two isomers were present in the ratio 1:0.8. Crystallization of this mixture of isomers from toluene provided one of the two isomers almost free of the other isomer. On the other hand, evaporation of the mother liquor and crystallization of the residue provided the other isomer almost free of the first one as shown by ^1^H-NMR experiments and comparing the spectra of the two isomers with the one received from their mixture. In an attempt to identify which isomer is which, we decided to proceed with further recrystallizations of the almost pure isomers, obtained as described above. To our delight, the second recrystallization of the former isomer provided it in a suitable crystalline form to allow for an X-ray analysis. Unsuccessful were, however, our attempts to obtain the second isomer in an also suitable crystalline form for X-ray analysis.

We now wish to report the results of the X-ray crystallographic analysis of the former isomer which allowed us to determine unambiguously its configuration around the exocyclic double bond. As it can be seen from Figure 3, this compound actually has the *Z* configuration around the double bond (isomer 5 b) and therefore the other isomer should be the E isomer (5a).

The molecule shows an almost planar arrangement of the ring system and the new double bond (the maximum deviation from their mean plane being 0.091 (2) Å for atom C10), whereas the carbonyl O2 atom lies 0.602 (3) Å outside the plane and forms a 45.1 (3)° dihedral angle with it. The C9═ C10 (*Csp*^2^–*Csp*^2^) distance of 1.340 (4) Å confirms the localization of the double bond at this position. Each molecule is linked to four neighbouring molecules *via *intermolecular hydrogen bonds between the amide H atoms and the two O atoms (N—H1N···O1 and N—H2N···O2) to form cyclic structures with *R*_4_^4^(24) graph-set motifs (Bernstein *et al.*., 1995). This bonding pattern results in a network of connected *R*_4_^4^(24) rings lying on pleated layers parallel to the *ab* plane (Table 1 and Fig. 4). The *tert*-butyl moieties are packed between the layers.

The linkage inside each layer is further supported by weak *π*—*π* stacking interactions among the central five-membered ring and one of the attached six-membered rings of the fluorene moieties. Specifically, the C1'-C4'-C5'-C8'-C9 fulvene ring at (x, y, z) and the C1'-C1-C2-C3-C4-C4' aryl ring at (1/2+x, y, 3/2-z) are almost parallel forming between them a dihedral angle of 6.5 (2)°. The centroid separation of the two rings is 3.583 (2) Å and the perpendicular distance of the first centroid on the plane of the second ring is 3.421 (1) Å, corresponding to a centroid offset of 1.07 (2) Å.

### Experimental

A mixture of 4-amino-9*H*-fluoren-9-one (0.78 g, 4 mmol) and ylide BCMP (3.16 g, 8.4 mmol) in anhydrous DMF (4 ml) was stirred at 100 *^o^*C for 4 days under an atmosphere of argon. The resulting solution was diluted with 30 ml e thylacetate (EtOAc) and then washed with H_2_O (3 *x* 10 ml). The organic layer was dried (Na_2_SO_4_) and evaporated to dryness under reduced pressure. The residue was subjected to flash column chromatography using as eluant the solvent system PhMe/EtOAc (9.5:0.5).

The fractions with *R*_f_ 0.3 in the same solvent system were pooled and evaporated to leave 1.1 g (95% yield) of pure compound in the form of a reddish oil. ^1^H NMR of the product revealed the presence of the two geometrical isomers in the ratio 1:0.8 calculated on the basis of the integration of the two peaks at 8.839 and 8.329 p.p.m. where proton H-8 resonates for the isomers 5a and 5 b, respectively.The product was dissolved in the minimum volume of hot toluene. The resulting solution was then left to attain ambient temperature and then cooled at 5 *^o^*C for 2 days. The crystalline precipitate was collected, washed with ice-cold toluene and dried under reduced pressure. It weighed 60 mg. ^1^H NMR of the crystals thus obtained showed them to be almost the pure isomer (5a:5 b=1:10) with H-8 resonating at 8.329 p.p.m.. The mother liquor from the crystallization was evaporated to dryness and the residue was crystallized also from toluene to provide 70 mg of the almost pure alternative isomer with H-8 resonating at 8.839 p.p.m.. Recrystallization of the first crystalline isomer finally gave reddish crystals suitable for X-ray analysis. This isomer had m.p. 386–387 K.

### Refinement

The amine H atoms and that attached to C14 were located in difference Fourier maps and their positions were refined freely along with *U*_iso_(H) equal to 1.5*U*_eq _and 1.2*U*_eq _of their parent atoms, respectively. The methyl H atoms were constrained to an ideal geometry [C—H = 0.96 Å and *U*_iso_(H) = 1.5*U*_eq_(C)], but were allowed to rotate freely about the C—C bonds. The remaining phenyl group H atoms were placed in geometrically idealized positions and constrained to ride on their parent C atoms [C—H = 0.93 Å and *U*_iso_(H) = 1.2*U*_eq_(C)]. Five low-angle reflections were omitted from the final cycles of refinement because their observed intensities were significantly lower than the calculated values, being apparently obscured by the beam stop.

### Crystal data

**Table:**

C_19_H_19_NO_2_	*F*(000) = 1248
*M**_r_* = 293.35	*D*_x_ = 1.272 Mg m^−^^3^
Orthorhombic, *P**b**c**a*	Mo *K*α radiation, λ = 0.71073 Å
Hall symbol: -P 2ac 2ab	Cell parameters from 3945 reflections
*a* = 9.0820 (12) Å	θ = 3.1–30.2°
*b* = 13.7330 (17) Å	µ = 0.08 mm^−^^1^
*c* = 24.568 (3) Å	*T* = 100 K
*V* = 3064.2 (7) Å^3^	Prism, light yellow
*Z* = 8	0.32 × 0.26 × 0.16 mm

### Data collection

**Table:**

Oxford Diffraction Xcalibur-3 with Sapphire CCD diffractometer	2671 independent reflections
Radiation source: Enhance (Mo) X-ray source	1671 reflections with *I* > 2σ(*I*)
graphite	*R*_int_ = 0.119
Detector resolution: 16.0288 pixels mm^-1^	θ_max_ = 25.0°, θ_min_ = 3.2°
ω and φ scans	*h* = −8→10
Absorption correction: multi-scan (CrysAlis RED; Oxford Diffraction, 2008)	*k* = −16→14
*T*_min_ = 0.956, *T*_max_ = 0.989	*l* = −27→29
18331 measured reflections	

### Refinement

**Table:**

Refinement on *F*^2^	Primary atom site location: structure-invariant direct methods
Least-squares matrix: full	Secondary atom site location: difference Fourier map
*R*[*F*^2^ > 2σ(*F*^2^)] = 0.063	Hydrogen site location: difference Fourier map
*wR*(*F*^2^) = 0.143	H atoms treated by a mixture of independent and constrained refinement
*S* = 1.01	*w* = 1/[σ^2^(*F*_o_^2^) + (0.069*P*)^2^] where *P* = (*F*_o_^2^ + 2*F*_c_^2^)/3
2671 reflections	(Δ/σ)_max_ < 0.001
211 parameters	Δρ_max_ = 0.30 e Å^−^^3^
0 restraints	Δρ_min_ = −0.22 e Å^−^^3^

### Special details

**Table:**

Experimental. IR (KBr, n, cm^-1^): 3404 and 3338 (*w*), 1700 (*s*), 1628 (*m*); ^1^H NMR (400 MHz, CDCl_3_): *d *8.329 (*d*, *J* = 7.6 Hz, 1H, H-8), 7.623 (*d*, *J* = 7.6 Hz, 1H, H-5), 7.528 (*d*, *J*= 7.6 Hz, 1H, H-1), 7.319 (*t*, *J* = 6.8 Hz, 1H, H-7), 7.152 (*t*, *J*= 6.8 Hz, 1H, H-6), 7.089 (*t*, *J* = 8 Hz, 1H, H-2), 6.707 (*d*, *J*= 7.6 Hz, 1H, H-3), 6.628 (*s*, 1H, H-10), 3.959 (*s*, 2H, NH_2_),1.541 (*s*, 9H, H-13) p.p.m.; EI—MS *m/z*: 293 (*M*^+^, 9), 237 (100), 220 (11), 193 (13), 180 (17), 165 (12).
Geometry. All e.s.d.'s (except the e.s.d. in the dihedral angle between two l.s. planes) are estimated using the full covariance matrix. The cell e.s.d.'s are taken into account individually in the estimation of e.s.d.'s in distances, angles and torsion angles; correlations between e.s.d.'s in cell parameters are only used when they are defined by crystal symmetry. An approximate (isotropic) treatment of cell e.s.d.'s is used for estimating e.s.d.'s involving l.s. planes.
Refinement. Refinement of *F*^2^ against ALL reflections. The weighted *R*-factor *wR* and goodness of fit *S* are based on *F*^2^, conventional *R*-factors *R* are based on *F*, with *F* set to zero for negative *F*^2^. The threshold expression of *F*^2^ > σ(*F*^2^) is used only for calculating *R*-factors(gt) *etc*. and is not relevant to the choice of reflections for refinement. *R*-factors based on *F*^2^ are statistically about twice as large as those based on *F*, and *R*- factors based on ALL data will be even larger.

### Fractional atomic coordinates and isotropic or equivalent isotropic displacement parameters (Å^2^)

**Table:**

	*x*	*y*	*z*	*U*_iso_*/*U*_eq_	
C1'	0.1196 (3)	0.06224 (19)	0.71979 (11)	0.0225 (7)	
C1	0.0627 (3)	0.11302 (19)	0.67557 (12)	0.0268 (7)	
H1	0.0906	0.0977	0.6402	0.032*	
C2	−0.0373 (3)	0.18742 (19)	0.68596 (12)	0.0301 (7)	
H2	−0.0751	0.2232	0.6570	0.036*	
C3	−0.0816 (3)	0.20922 (19)	0.73829 (12)	0.0275 (7)	
H3	−0.1496	0.2589	0.7437	0.033*	
C4	−0.0270 (3)	0.15855 (19)	0.78340 (12)	0.0236 (7)	
C4'	0.0775 (3)	0.08588 (18)	0.77339 (11)	0.0225 (7)	
C5'	0.1565 (3)	0.02216 (19)	0.81212 (12)	0.0225 (7)	
C5	0.1598 (3)	0.0176 (2)	0.86812 (12)	0.0271 (7)	
H5	0.1017	0.0592	0.8888	0.033*	
C6	0.2509 (3)	−0.0500 (2)	0.89319 (12)	0.0315 (8)	
H6	0.2537	−0.0534	0.9310	0.038*	
C7	0.3378 (3)	−0.1125 (2)	0.86264 (12)	0.0294 (7)	
H7	0.3974	−0.1579	0.8801	0.035*	
C8	0.3365 (3)	−0.1077 (2)	0.80637 (11)	0.0248 (7)	
H8	0.3956	−0.1492	0.7859	0.030*	
C8'	0.2461 (3)	−0.04057 (18)	0.78088 (11)	0.0216 (7)	
C9	0.2258 (3)	−0.02063 (19)	0.72239 (12)	0.0224 (7)	
C10	0.2879 (3)	−0.0755 (2)	0.68355 (12)	0.0241 (7)	
H10	0.350 (3)	−0.129 (2)	0.6933 (11)	0.029*	
C11	0.2785 (3)	−0.0635 (2)	0.62405 (12)	0.0258 (7)	
C12	0.2695 (3)	−0.1621 (2)	0.54059 (11)	0.0268 (7)	
C13	0.2656 (4)	−0.2720 (2)	0.53364 (13)	0.0450 (9)	
H13A	0.3497	−0.3003	0.5512	0.067*	
H13B	0.2675	−0.2878	0.4956	0.067*	
H13C	0.1772	−0.2974	0.5497	0.067*	
C14	0.4087 (4)	−0.1212 (3)	0.51722 (13)	0.0445 (9)	
H14A	0.4062	−0.0514	0.5194	0.067*	
H14B	0.4175	−0.1406	0.4798	0.067*	
H14C	0.4915	−0.1453	0.5374	0.067*	
C15	0.1325 (4)	−0.1164 (2)	0.51698 (13)	0.0421 (9)	
H15A	0.0472	−0.1425	0.5349	0.063*	
H15B	0.1272	−0.1304	0.4787	0.063*	
H15C	0.1358	−0.0472	0.5223	0.063*	
N	−0.0702 (3)	0.18283 (18)	0.83630 (11)	0.0298 (6)	
H1N	−0.147 (4)	0.218 (2)	0.8360 (13)	0.045*	
H2N	−0.095 (3)	0.132 (2)	0.8587 (13)	0.045*	
O1	0.2699 (2)	−0.15191 (12)	0.60048 (7)	0.0262 (5)	
O2	0.2815 (3)	0.01219 (14)	0.59924 (8)	0.0384 (6)	

### Atomic displacement parameters (Å^2^)

**Table:**

	*U*^11^	*U*^22^	*U*^33^	*U*^12^	*U*^13^	*U*^23^
C1'	0.0268 (16)	0.0110 (14)	0.0297 (17)	−0.0066 (12)	−0.0013 (13)	−0.0008 (12)
C1	0.0332 (17)	0.0165 (15)	0.0306 (17)	−0.0016 (13)	−0.0030 (14)	0.0037 (13)
C2	0.0370 (19)	0.0179 (15)	0.0353 (18)	−0.0032 (14)	−0.0047 (15)	0.0060 (14)
C3	0.0291 (18)	0.0108 (15)	0.043 (2)	0.0003 (13)	−0.0027 (15)	−0.0014 (13)
C4	0.0264 (16)	0.0130 (14)	0.0314 (18)	−0.0047 (12)	0.0012 (14)	−0.0025 (12)
C4'	0.0288 (16)	0.0111 (14)	0.0277 (17)	−0.0044 (12)	−0.0031 (13)	0.0004 (12)
C5'	0.0263 (16)	0.0124 (14)	0.0289 (17)	−0.0047 (12)	−0.0032 (14)	−0.0029 (12)
C5	0.0349 (18)	0.0194 (15)	0.0270 (17)	−0.0007 (13)	0.0022 (14)	−0.0055 (13)
C6	0.0423 (19)	0.0264 (17)	0.0259 (17)	−0.0013 (15)	−0.0006 (15)	−0.0039 (13)
C7	0.0369 (18)	0.0204 (16)	0.0310 (18)	0.0022 (14)	−0.0043 (15)	−0.0008 (14)
C8	0.0316 (17)	0.0146 (14)	0.0281 (17)	−0.0009 (13)	−0.0018 (14)	−0.0051 (13)
C8'	0.0296 (16)	0.0109 (13)	0.0243 (16)	−0.0051 (12)	−0.0001 (14)	−0.0038 (12)
C9	0.0230 (16)	0.0126 (14)	0.0317 (17)	−0.0043 (12)	−0.0033 (14)	−0.0004 (12)
C10	0.0329 (18)	0.0107 (14)	0.0287 (17)	−0.0011 (12)	−0.0011 (14)	−0.0008 (13)
C11	0.0332 (18)	0.0153 (15)	0.0288 (16)	−0.0029 (13)	0.0013 (14)	−0.0008 (13)
C12	0.0359 (18)	0.0220 (16)	0.0225 (16)	−0.0015 (14)	0.0017 (14)	−0.0009 (12)
C13	0.083 (3)	0.0263 (18)	0.0259 (18)	−0.0044 (18)	0.0041 (18)	−0.0059 (14)
C14	0.052 (2)	0.053 (2)	0.0285 (18)	−0.0116 (18)	0.0043 (17)	0.0036 (16)
C15	0.049 (2)	0.046 (2)	0.0303 (18)	0.0002 (17)	−0.0025 (17)	−0.0051 (16)
N	0.0346 (16)	0.0172 (14)	0.0374 (16)	0.0019 (12)	0.0056 (13)	−0.0018 (12)
O1	0.0446 (13)	0.0125 (10)	0.0214 (11)	−0.0021 (9)	0.0032 (10)	−0.0001 (8)
O2	0.0723 (17)	0.0127 (10)	0.0303 (12)	−0.0024 (10)	−0.0025 (11)	0.0043 (9)

### Geometric parameters (Å, °)

**Table:**

C1'—C1	1.391 (4)	C8'—C9	1.475 (4)
C1'—C4'	1.409 (4)	C9—C10	1.340 (4)
C1'—C9	1.493 (4)	C10—C11	1.474 (4)
C1—C2	1.391 (4)	C10—H10	0.96 (3)
C1—H1	0.9300	C11—O2	1.205 (3)
C2—C3	1.380 (4)	C11—O1	1.347 (3)
C2—H2	0.9300	C12—O1	1.478 (3)
C3—C4	1.399 (4)	C12—C14	1.498 (4)
C3—H3	0.9300	C12—C15	1.510 (4)
C4—N	1.398 (4)	C12—C13	1.519 (4)
C4—C4'	1.400 (4)	C13—H13A	0.9600
C4'—C5'	1.478 (4)	C13—H13B	0.9600
C5'—C5	1.377 (4)	C13—H13C	0.9600
C5'—C8'	1.412 (4)	C14—H14A	0.9600
C5—C6	1.387 (4)	C14—H14B	0.9600
C5—H5	0.9300	C14—H14C	0.9600
C6—C7	1.387 (4)	C15—H15A	0.9600
C6—H6	0.9300	C15—H15B	0.9600
C7—C8	1.384 (4)	C15—H15C	0.9600
C7—H7	0.9300	N—H1N	0.85 (3)
C8—C8'	1.384 (4)	N—H2N	0.92 (3)
C8—H8	0.9300		
			
C1—C1'—C4'	120.9 (3)	C10—C9—C1'	132.1 (3)
C1—C1'—C9	131.0 (3)	C8'—C9—C1'	105.3 (2)
C4'—C1'—C9	108.1 (2)	C9—C10—C11	128.3 (3)
C1'—C1—C2	117.9 (3)	C9—C10—H10	120.2 (16)
C1'—C1—H1	121.1	C11—C10—H10	111.5 (16)
C2—C1—H1	121.1	O2—C11—O1	124.1 (3)
C3—C2—C1	121.4 (3)	O2—C11—C10	126.6 (3)
C3—C2—H2	119.3	O1—C11—C10	109.2 (2)
C1—C2—H2	119.3	O1—C12—C14	110.1 (2)
C2—C3—C4	121.8 (3)	O1—C12—C15	110.2 (2)
C2—C3—H3	119.1	C14—C12—C15	113.1 (3)
C4—C3—H3	119.1	O1—C12—C13	101.9 (2)
N—C4—C3	121.2 (3)	C14—C12—C13	110.5 (3)
N—C4—C4'	121.6 (3)	C15—C12—C13	110.6 (3)
C3—C4—C4'	117.1 (3)	C12—C13—H13A	109.5
C4—C4'—C1'	120.8 (3)	C12—C13—H13B	109.5
C4—C4'—C5'	129.7 (3)	H13A—C13—H13B	109.5
C1'—C4'—C5'	109.5 (2)	C12—C13—H13C	109.5
C5—C5'—C8'	120.2 (3)	H13A—C13—H13C	109.5
C5—C5'—C4'	132.9 (3)	H13B—C13—H13C	109.5
C8'—C5'—C4'	106.9 (2)	C12—C14—H14A	109.5
C5'—C5—C6	119.1 (3)	C12—C14—H14B	109.5
C5'—C5—H5	120.4	H14A—C14—H14B	109.5
C6—C5—H5	120.4	C12—C14—H14C	109.5
C5—C6—C7	120.9 (3)	H14A—C14—H14C	109.5
C5—C6—H6	119.6	H14B—C14—H14C	109.5
C7—C6—H6	119.6	C12—C15—H15A	109.5
C8—C7—C6	120.4 (3)	C12—C15—H15B	109.5
C8—C7—H7	119.8	H15A—C15—H15B	109.5
C6—C7—H7	119.8	C12—C15—H15C	109.5
C8'—C8—C7	119.3 (3)	H15A—C15—H15C	109.5
C8'—C8—H8	120.4	H15B—C15—H15C	109.5
C7—C8—H8	120.4	C4—N—H1N	111 (2)
C8—C8'—C5'	120.1 (3)	C4—N—H2N	116 (2)
C8—C8'—C9	129.7 (2)	H1N—N—H2N	104 (3)
C5'—C8'—C9	110.1 (2)	C11—O1—C12	120.9 (2)
C10—C9—C8'	122.4 (3)		
			
C4'—C1'—C1—C2	−0.3 (4)	C7—C8—C8'—C5'	0.0 (4)
C9—C1'—C1—C2	179.3 (3)	C7—C8—C8'—C9	179.4 (3)
C1'—C1—C2—C3	−1.4 (4)	C5—C5'—C8'—C8	0.8 (4)
C1—C2—C3—C4	0.8 (4)	C4'—C5'—C8'—C8	178.5 (2)
C2—C3—C4—N	178.5 (3)	C5—C5'—C8'—C9	−178.8 (2)
C2—C3—C4—C4'	1.4 (4)	C4'—C5'—C8'—C9	−1.0 (3)
N—C4—C4'—C1'	179.9 (2)	C8—C8'—C9—C10	6.5 (5)
C3—C4—C4'—C1'	−3.1 (4)	C5'—C8'—C9—C10	−174.0 (3)
N—C4—C4'—C5'	1.6 (4)	C8—C8'—C9—C1'	−177.6 (3)
C3—C4—C4'—C5'	178.7 (3)	C5'—C8'—C9—C1'	1.9 (3)
C1—C1'—C4'—C4	2.6 (4)	C1—C1'—C9—C10	−6.4 (5)
C9—C1'—C4'—C4	−177.1 (2)	C4'—C1'—C9—C10	173.2 (3)
C1—C1'—C4'—C5'	−178.8 (2)	C1—C1'—C9—C8'	178.3 (3)
C9—C1'—C4'—C5'	1.5 (3)	C4'—C1'—C9—C8'	−2.0 (3)
C4—C4'—C5'—C5	−4.5 (5)	C8'—C9—C10—C11	−179.2 (3)
C1'—C4'—C5'—C5	177.1 (3)	C1'—C9—C10—C11	6.2 (5)
C4—C4'—C5'—C8'	178.1 (3)	C9—C10—C11—O2	39.7 (5)
C1'—C4'—C5'—C8'	−0.3 (3)	C9—C10—C11—O1	−142.0 (3)
C8'—C5'—C5—C6	−0.7 (4)	O2—C11—O1—C12	2.5 (4)
C4'—C5'—C5—C6	−177.8 (3)	C10—C11—O1—C12	−175.8 (2)
C5'—C5—C6—C7	−0.1 (4)	C14—C12—O1—C11	60.3 (3)
C5—C6—C7—C8	0.8 (5)	C15—C12—O1—C11	−65.1 (3)
C6—C7—C8—C8'	−0.7 (4)	C13—C12—O1—C11	177.6 (2)

### Hydrogen-bond geometry (Å, °)

**Table:**

*D*—H···*A*	*D*—H	H···*A*	*D*···*A*	*D*—H···*A*
N—H1N···O1^i^	0.85 (3)	2.62 (3)	3.295 (3)	137 (3)
N—H2N···O2^ii^	0.92 (3)	2.24 (3)	3.133 (3)	163 (3)

Symmetry codes: (i) −*x*, *y*+1/2, −*z*+3/2; (ii) *x*−1/2, *y*, −*z*+3/2.
